# A new mode of luminescence in lanthanide oxalates metal–organic frameworks

**DOI:** 10.1038/s41598-022-23658-z

**Published:** 2022-11-05

**Authors:** Reem H. Alzard, Lamia A. Siddig, Na’il Saleh, Ha L. Nguyen, Quynh Anh T. Nguyen, Thi H. Ho, Viet Q. Bui, K. Sethupathi, P. K. Sreejith, Ahmed Alzamly

**Affiliations:** 1 Department of Chemistry, UAE University, P.O. Box 15551, Al-Ain, UAE; 2 Department of Chemistry, University of California Berkeley, Berkeley, CA 94720 USA; 3 Kavli Energy Nanoscience Institute at UC Berkeley, Berkeley, CA 94720 USA; 4 Berkeley Global Science Institute, Berkeley, CA 94720 USA; 5 Joint UAEU–UC Berkeley Laboratories for Materials Innovations, United Arab Emirates University, 15551, Al-Ain, UAE; 6 Advanced Institute of Science and Technology, The University of Danang, 41 Le Duan, Danang, Vietnam; 7 Low Temperature Physics Laboratory, Department of Physics, Indian Institute of Technology Madras, Chennai, 600036 India

**Keywords:** Chemistry, Materials chemistry, Metal-organic frameworks

## Abstract

Two lanthanide metal–organic frameworks [Ln-MOFs, Ln = Eu(III), Tb(III)] composed of oxalic acid and Ln building units were hydrothermally synthesized and fully characterized by powder X-ray diffraction, Fourier-transform infrared spectroscopy, thermogravimetric analysis, scanning electron microscope, and energy-dispersive X-ray spectroscopy. Furthermore, their magnetic susceptibility measurements were obtained using SQUID based vibrating sample magnetometer (MPMS 3, Quantum Design). Both Ln-MOFs exhibited highly efficient luminescent property. Solid-state photoluminescence (PL) measurements revealed phosphorescence emission bands of Eu-MOF and Tb-MOF centered at 618 nm (red emission) and 550 nm (green emission) upon excitation at 396 nm and 285 nm, respectively. Eu-MOF and Tb-MOF displayed a phosphorescence quantum yield of 53% and 40%, respectively. Time-resolved PL analyses showed very long lifetime values, at 600 and 1065 ± 1 µs for Eu-MOF and Tb-MOF, respectively. Calculations performed by density functional theory indicated a charge transfer form metal centres to the ligand which was in good agreement with the experimental studies. Therefore, this new mode of highly photoluminescent MOF materials is studied for the first time which paves the way for better understanding of these systems for potential applications.

## Introduction

Unlike d-block transition metals that have been mainly used to construct metal–organic framework (MOFs), lanthanide (Ln) metals are larger in radius, higher in coordination number, and have unique electronic configuration of partially filled 4f orbitals^1^. Particularly, Ln metals with their attractive physical properties when constructed MOFs give rise to varied coordination geometry^2^ that endows specific luminescent characteristics including bright visible emission, long decay lifetime^3^, and large Stokes shifts^4^. These characteristics allow the diverse employment of luminescence Ln-MOFs in catalytic^5,6^, optical^7,8^, medical^9,10^, and magnetic applications^11^. Therefore, using Ln metals as trivalent cations in designing and synthesizing MOFs has been of great interest in recent years^2^.

As Ln metals are classically hard acids, they have high binding affinity to hard base donor atoms like oxygen or mixed atoms of oxygen and nitrogen. Polycarboxylate linkers are thus commonly used to construct Ln-MOFs^12–14^. Specifically, MOFs based on Ln oxalates have been studied due to their interesting optical and magnetic properties^15^ which are employed in chemical thermal analysis^16^ and nuclear chemistry^17^. For applying photoluminescent properties of Ln^3+^, several strategies have been approached to enhance their weak light absorption using different organic linkers for the antenna effect^18^. Polycarboxylate linkers for example allow the sensitization process to start from the lowest singlet state, S_1_, to T_1_ and finally to Ln^3+^ ions^19,20^ which eventually leads to enhance the MOF photoluminescent properties. Nonetheless, it was possible to switch on phosphorescence in the absence of ligand-to-metal energy transfer process as already observed in simple organic molecules^21^ or upon coordination of tris-chelates organic sensitizer, as reported for La^3+^, Gd^3+^, or Lu^3+^ ions^22^.

Recently, researchers have been focusing on developing solid-state phosphorescent materials^23,24^. However, studies on MOFs for phosphorescence at room temperature are very rare. Literature shows that zinc isophthalate MOF (Y346)^25^ and zeolitic imidazolate framework-8 (ZIF-8; ZIFs is a subclass of MOFs)^26^ encapsulated with Rhodamine B dye and coronene fluorophore, respectively, are two MOF materials that display the enhanced phosphorescent emission. Surprisingly, phosphorescent properties of Ln-oxalates have not been investigated. Our motivation is to utilize oxalic acid that exbibits different binding modes to Ln metal ions and further study the resulting distinctive properties of the MOF having various dimensional structures (2D and 3D). Particularly, after a literature search and screening, we choose and have successfully reproduced two Ln-MOFs (Ln = Eu and Tb)^27^ and found that they exhibit interesting photoluminescent property. These are the first luminescent Ln oxalates that showed high phosphorescence quantum yields and very long phosphorescence lifetime compared to similar reported systems^13,14,28^. The solid-state photoluminescent intensity and the emission lifetime behaviour of Eu- and Tb-oxalate have been experimentally analysed and theoretically calculated using time-dependent density functional theory (TD-DFT). The bridging modes and flexibility of oxalic acid makes it interesting in the MOF systems, producing new materials with variant multidimensional structures and physical properties (e.g., optical, magnetic, conductive, and more to be explored)^29^. These findings provide insight into unique photophysical processes related to the triplet state of Ln-oxalates and possibly open a new pathway for phosphorescent applications in many fronts such as optics and sensing.

## Experimental section

### Materials and general procedures

Europium(III) nitrate hydrate Eu(NO_3_)_3_⋅H_2_O, terbium(III) nitrate pentahydrate Tb(NO_3_)_3_⋅5H_2_O, oxalic acid dihydrate (COOH)_2_⋅2H_2_O, and anhydrous dimethylformamide (DMF) were purchased from Sigma-Aldrich and used without further purification.

### Synthesis of Ln-MOFs

Eu-MOF and Tb-MOF were prepared according to the previously reported procedure with significant modifications^27,30^. In a typical preparation, 2.5 mmol (315 mg) of oxalic acid was firstly dissolved in 5 mL DMF, and 1 mmol of the Ln-nitrate salt (337.98 mg of Eu-nitrate or 435.02 mg of Tb-nitrate) was dissolved in 5 mL deionized water. Solutions were mixed in a 23 mL Teflon-lined Parr autoclave and placed in a preheated oven at 120 °C for 3 days. The reaction was subsequently cooled down to an ambient temperature and a white powder was obtained. The product was transferred to a scintillation vial, washed and soaked in deionized water for 1 h. The solvent was decanted; the product was collected, dried, and activated at 100 °C under vacuum for 2 h.

## Characterization

### Powder X-ray diffraction (PXRD)

PXRD patterns of Ln-MOFs were recorded on a Rigaku MiniFlex benchtop X-ray diffractometer using CuKα radiation tube (λ = 1.542 Å) at 40 kV along the range of 3°–50° 2θ with a rate of 2° min^−1^.

### Fourier-transform infrared (FT-IR) spectroscopy

Both Ln-oxalate MOFs were characterized using Agilent Cary 600 Series FT-IR Spectrometer with ATR-IR spectroscopy. The spectral data were recorded in the range of 4000 to 500 cm^−1^ and the average of 512 scans were calculated for each spectrum with a 2 cm^−1^ spectral resolution. The MOF spectra were automatically subtracted from the background spectrum that was recorded first.

### Thermogravimetric analysis (TGA)

Shimadzu TGA-50 analyser was used to conduct TGA under nitrogen flow at a rate of 100 mL min^−1^. The chamber heating flow was 5 °C min^−1^ where the activated Ln-MOF sample was placed in an aluminium pan holder.

### Scanning electron microscope (SEM) and energy-dispersive X-ray spectroscopy (EDX)

SEM images were captured at high vacuum, accelerating voltage of 30 kV and a magnification of 5000 × using Quattro SEM instrument. The instrument was equipped with an energy-dispersive X-ray detector for the EDX analysis.

### Photoluminescence (PL) spectra and lifetime measurements

The PL spectra of the MOF samples were recorded by using FS5 spectrofluorometer (Edinburgh instrument, Edinburgh, UK) which utilized a continuous xenon lamp to excite the sample at 285, 375, and 396 nm. The corresponding excitation spectra were monitored at 618 and 452 nm. The time-resolved PL measurements were conducted using the same instrument equipped with a pulsed flash lamp with a repetition rate of 100 Hz (pulsed period 10 ms) and a pulse width of ~ 400 ns. The data were recorded by using multi-channel scaling as a single photon counting technique. The PL decays were collected at 618 and 542 nm with a total count rate of 10,000 counts s^−1^ by using a temperature stabilised photomultiplier tube detector (R928P, Hamamatsu, Japan). The data were fitted to a single exponential model function utilizing tail fitting procedure and Least-Square Statistical Analysis (Chi-square and residual plot) to assess the goodness of fit. The single lifetime (*τ*_*i*_) was extracted with an estimated experimental error of 2%. Cell holders with front-face geometries (SC-10) for measurements in the solid state on both FS5.

### Absolute PL quantum yield (QY) measurements

Absolute QY were estimated for the solid samples on the FS5 spectrometer by utilizing the continuous xenon lamp and an integrating sphere (SC-30) and by comparing the measured direct and indirect signals from the sample to that generated from the PTFE reference through direct excitation. The bandwidths were kept at 3 and 0.25 for the excitation and emission monochromators. The sample excited at 285, 375 and 396 nm. The error is 2% of the estimated experimental value.

### Magnetic and temperature dependent studies

Magnetic properties of Tb MOF and Eu-MOF were characterized using the superconducting quantum interference device analysis (SQUID) based vibrating sample magnetometer (MPMS 3, Quantum Design) operating between 5 and 300 K. The temperature dependent direct current (DC) magnetization measurements have been carried out for Tb-MOF and Eu-MOF at an applied field of 1000 Oe and 5 quadrant magnetic field dependent DC magnetization measurements were done with an applied field of ranging from − 70 to + 70 kOe.

## Results and discussion

### Powder X-ray diffraction (PXRD)

**Figure 1:**
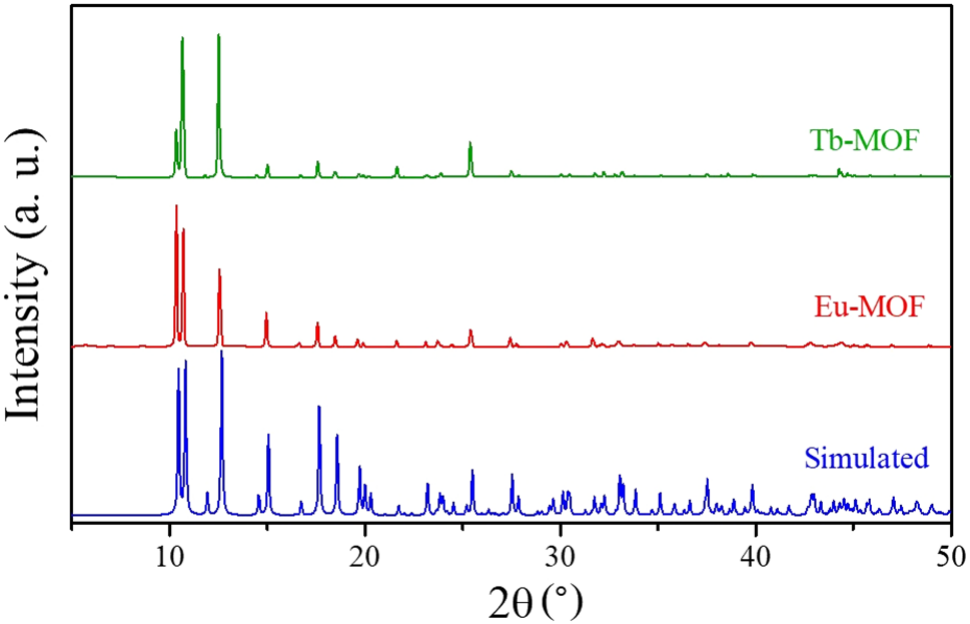
PXRD patterns of the prepared Ln-MOFs compared to the simulated PXRD pattern.

The freshly prepared and activated Ln-MOF samples were measured by PXRD to confirm the crystalline structures. The phase purity of the two Ln-MOFs was proven by comparing their simulated PXRD patterns^27,30^ (Fig. 1). The obtained diffraction patterns have proven the successful synthesis of isostructural frameworks of Eu-MOF and Tb-MOF which have the chemical formula of [Me_2_NH_2_][Ln(ox)_2_]·3H_2_O (Ln = Eu, Tb and ox = oxalic acid). Both Ln-MOFs crystallized in the orthorhombic lattice with unit cell parameters of *a* = 12.606 Å, *b* = 12.000 Å, and *c* = 12.686 Å. The morphology of the prepared MOFs is diamond-like crystals of ~ 5–10 µm as evidenced by SEM images (Supplementary Fig. S1). EDX analysis, on the other hand (Supplementary Figs. S2 and S3) confirmed the presence of Eu and Tb atoms in the synthesized Ln-MOFs (Supplementary Tables S1 and S2).

### Fourier-transform infrared (FT-IR) spectroscopy

**Figure 2:**
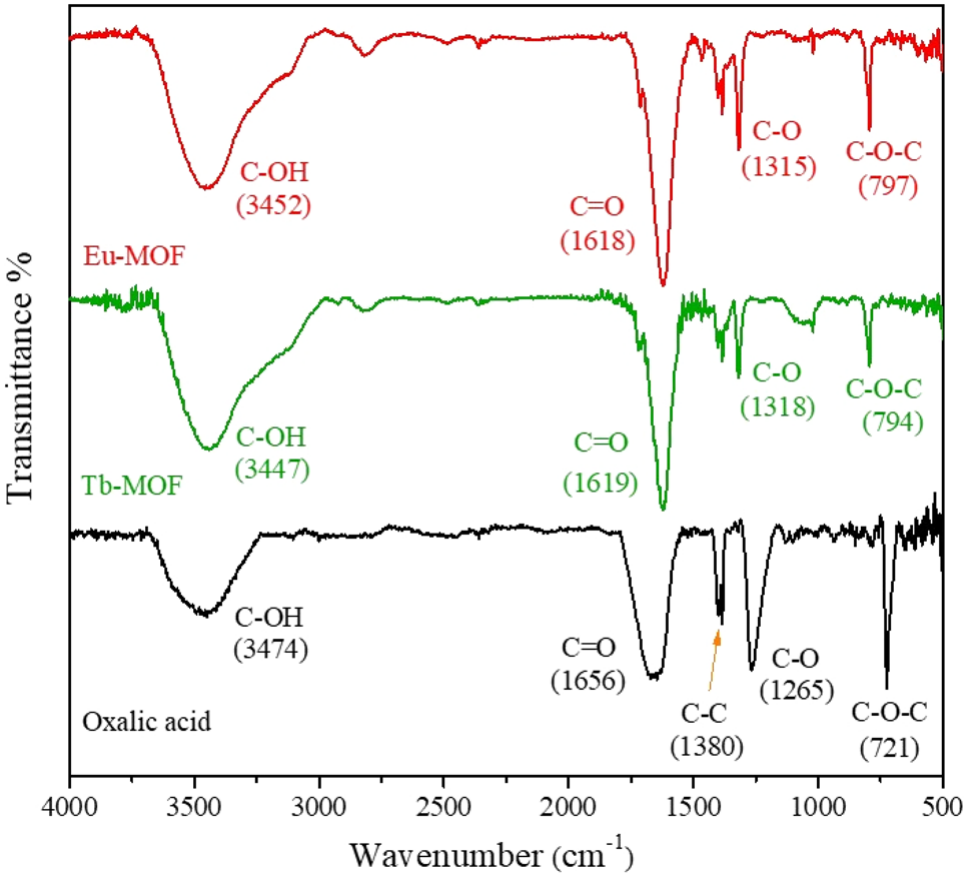
FT-IR spectra of Ln-MOFs with their assigned peaks.

The solid-state interaction and the binding mode of oxalic acid with Ln metals were characterized by FT-IR spectroscopy, as shown in Fig. 2. Comparing the FT-IR spectra of Ln-MOFs with the linker, a clear shift to a lower wavenumber of carboxylic acid peaks indicated the coordination between carbonyl groups and the lanthanide ions (1656 cm^−1^ for oxalic acid to 1618 cm^−1^ for Eu-MOF and 1919 cm^−1^ for Tb-MOF). Another shift was also observed at 1315 cm^−1^ and 1318 cm^−1^ for Eu-MOF and Tb-MOF, respectively, which corresponds to the stretch of C–O bonds. Moreover, the shift of the O–C–O band from 721 (oxalic acid) to 797 cm^−1^ (Eu-MOF) and 794 cm^−1^ (Tb-MOF) is a part of the binding site, which further supports the chelation of the carboxylate groups to the metal sites. C–C of oxalic acid (at 1380 cm^−1^), on the other hand, remains the same in all the FT-IR spectra.

### Thermogravimetric analysis (TGA)

The TGA profiles of Ln-MOFs were carried out from 25 to 600 °C under N_2_ atmosphere. Both Ln-MOFs have experienced similar thermal weight loss. As illustrated in supplementary Fig. S4, around 16% to 18% weight loss in step one starting at 106 °C indicated the loss of trapped coordinated water molecules from the lattice. Followed by step two, a dramatic weight loss was observed at 330 °C (40.1%) and 340 °C (30.8%) for Eu-MOF and Tb-MOF, respectively. Since Eu metal has a weaker binding affinity towards carboxylic group than Tb metal, Eu-MOF started to decompose before Tb-MOF as clearly shown in Fig. S4. The framework decomposition of both Ln-MOFs was attributed to the formation of a body-centered cubic form of Ln_2_O_3_^31^ that was also physically observed by the colour change of the samples after being heated.

### Photoluminescence (PL)

**Figure 3:**
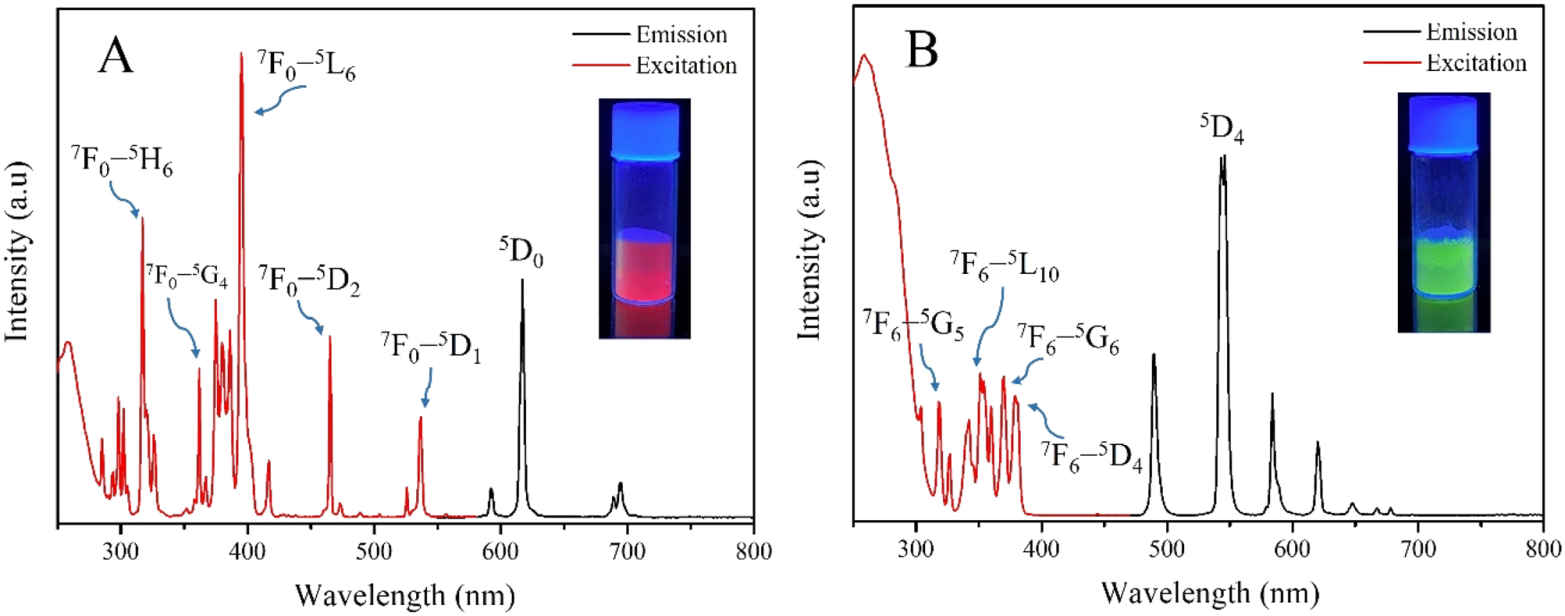
Excitation and emission spectra of the solid-state Eu-MOF (**A**) and Tb-MOF (**B**), along with their emissions at 396 and 285 nm excitations, respectively.

The absorption and the emission spectral properties of Ln-MOFs were measured using PL spectroscopy (Fig. 3). The absorption peaks of the metal ions indicated the Laporte-forbidden 4f–4f transitions in Ln metals^32^ that were assigned from the ground states of ^7^F_0_ and ^7^F_6_ for Eu(III) and Tb(III), respectively (Fig. 3). Upon excitation, Eu(III) exhibited three characteristic emission bands at 591 nm, 618 nm, and 695 nm (Fig. 3A) arose from the absorption transitions of ^7^F_0_–^5^H_6_, ^7^F_0_–^5^L_6_, and ^7^F_0_–^5^D_2_, respectively. Among these peaks, the emission at 618 nm is the most intense one that causes the red emission by Eu-MOF. This band could be attributed to the emission transition of ^5^D_0_–^7^F_2_^33–35^. On the other hand, Tb(III) exhibited four emission bands at 490 nm, 550 nm, 583 nm, and 620 nm that were assigned from the absorption transitions of ^7^F_6_–^5^G_5_, ^7^F_6_–^5^L_10_, ^7^F_6_–^5^G_6,_ and ^7^F_6_–^5^D_4_, respectively (Fig. 3B). The intense green luminescent of Tb-MOF is because of the strongest emission band at λ = 550 nm which originates from the ^5^D_4_–^7^F_5_ emission transition as shown in Fig. 3B^33–35^. Comparing the red and the green emissions exhibited by the two metals, Tb-MOF emission appears at lower wavelength than the emission of Tb-MOF (550 nm vs. 618 nm). Tb(III) ions emit from higher energy levels than Eu(III), thus, radiation is expected to appear at lower wavelength^36^. The characteristic excitation spectra of each metal (Fig. 3, red spectra) are different from each other which strongly denies the process found in luminescent MOF systems in which charge is usually transferred from ligand to the metal^37–39^. Moreover, if ligand-to-metal charge transfer exists in this mode, the excitation spectra of oxalic acid will appear in both Ln-MOFs instead.

The solid-state quantum yields were determined along with the lifetime decay using the integrating sphere method (Supplementary Fig. S5). According to Table 1, Eu-MOF has exhibited a quantum yield value of 53% when it was excited at λ = 375 nm or at 396 nm, while Tb-MOF has exhibited 40% upon excitation at λ = 285 nm. Lifetime data have revealed one exponential lifetime decay for each MOF which are 600 µs and 1057 µs for Eu-MOF and Tb-MOF, respectively. The currently prepared Ln-MOFs have the highest phosphorescence quantum yield values as stand-alone materials compared to other luminescent MOFs which were encapsulated by other luminescent compounds^25,26^. Detailed theoretical calculations have been conducted in order to understand why the two Ln-MOFs demonstrate higher quantum yields.

**Table 1:** Photophysical parameters of Ln-MOFs in the solid state.

Property	Eu-MOF	Tb-MOF
PL quantum yield, *ϕ*_*i*_ (%)	53	40
Lifetime decay, *τ*_*i*_ (µs)	600	1057
Chi-Square, *χ*^2^	1.154	1.188

### Time-resolved PL analysis

**Figure 4:**
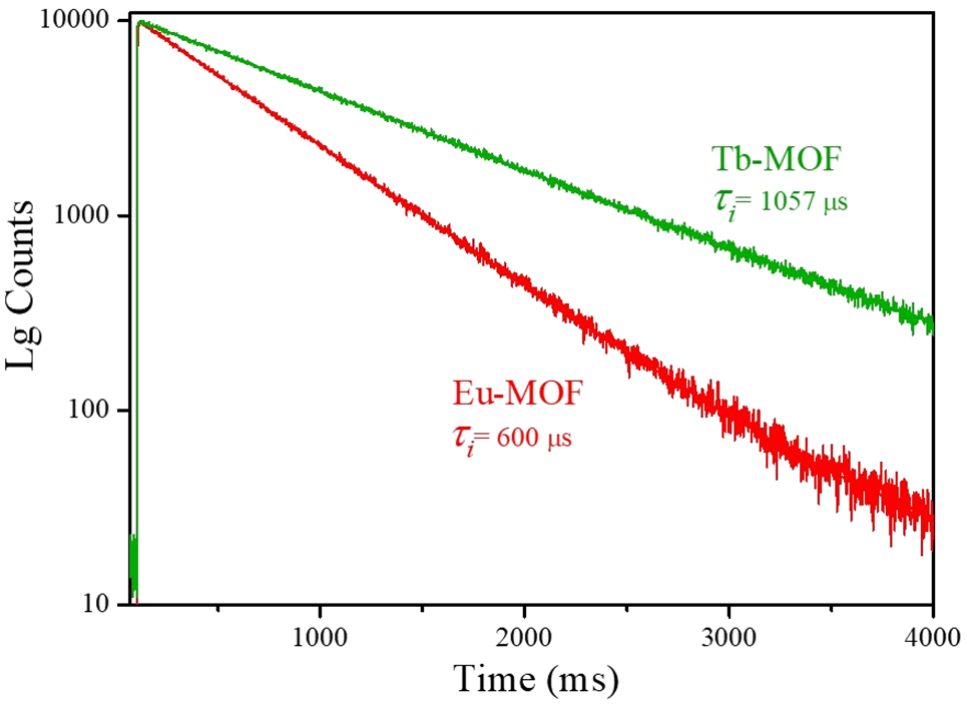
Time-resolved emission decays of Eu-MOF (λ_ex_ = 396 nm) and Tb-MOF (λ_ex_ = 285 nm) with their corresponding lifetime values.

The lifetime decays in fluorescent systems are normally due to the S_1_ → S_0_ transition which are usually fast^40^. However, as shown in Fig. 4 and Table 1, the observed long-lived lifetime species of both MOFs showed a phosphorescence relaxation from the T_1_ to the S_0_ levels. The Tb-MOF lifetime was shown to have a monoexponential decay longer than that in the Eu-MOF (1057 µs vs. 600 ± 1 µs). On the other hand, both Eu and Tb-MOFs have predominantly exhibited longer lifetimes than the recently reported values^14,41,42^ without modifying the structure with external fluorophores.

One of the factors that enhances a molecular photoluminescence intensity and extend the lifetime is the rigidity of the structure^37^. It is found in literature that luminescent MOFs are usually made from rigid aromatic linkers to achieve high fluorescence properties with short lifetimes^37,40^. However, our synthesized Ln-MOFs, composed of a simple, flexible diprotic oxalic acid, exhibited high phosphorescence quantum yields with longer lifetimes. The ability of the reported pristine Ln-MOFs which allow the phosphorescence radiative process to predominantly occur over fluorescence is a greater advantage than mixing ligands that are usually used to enhance the emission as found in literature. Herein, the unique photoluminescent properties of Ln-MOFs were successfully investigated and achieved which make lanthanide oxalate MOFs of a great interest for multiple phosphorescence applications such as optics and sensing.

### Theoretical and computational analysis

**Figure 5:**
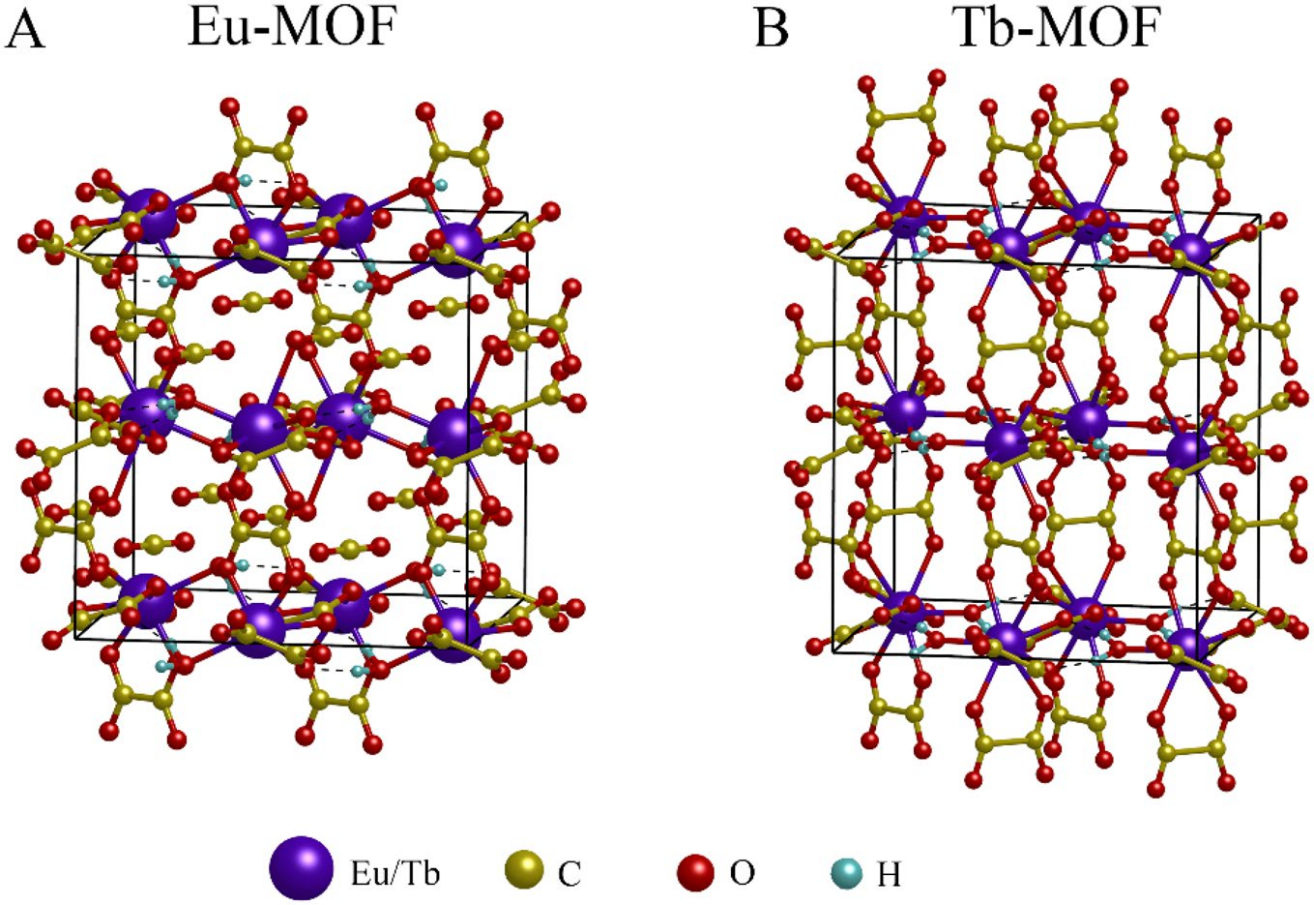
Eu-MOF (**A**) and Tb-MOF (**B**) crystal structure optimized by DFT calculation. Purple sphere denotes Eu or Tb; yellow, red, and cyan spheres denote C, O, and H, respectively.

Density functional theory (DFT) and time-dependent density functional theory (TDDFT) have been employed to study the structural, electronic, and optical properties of Eu-MOF and Tb-MOF. At the starting point, the optimization and electronic structure are performed using the Vienna Ab Initio Simulation Package (VASP)^43^ implemented the pseudopotential projector augmented-plane wave (PAW) method^44^. The generalized gradient approximation (GGA) parameterized by Perdew, Becke, and Ernzerhof (PBE) is adopted for the exchange–correlation potential^45^. A cut-off energy of 450 eV is used for wave function expansion, and a *k-*mesh of 3 × 3 × 3 is chosen for Brillouin zone integration. The crystal structures of Eu-MOF and Tb-MOF are constructed within orthorhombic framework based on experimental data, as respectively shown in Fig. 5. The unit cell and atomic coordinates are fully optimized with the force criterion of 1 meV/Å.

The optimized structures of Eu-MOF and Tb-MOF are used in the TDDFT calculations, performed by turbo TDDFT^46,47^ under Quantum Espresso Package^48^. Local density approximation (LDA)^49^ is used for exchange–correlation functional in the framework of the norm-conserving pseudopotential^50^. The cut-off energy is chosen as 476 eV with the Γ point-only *k*-mesh.

**Figure 6:**
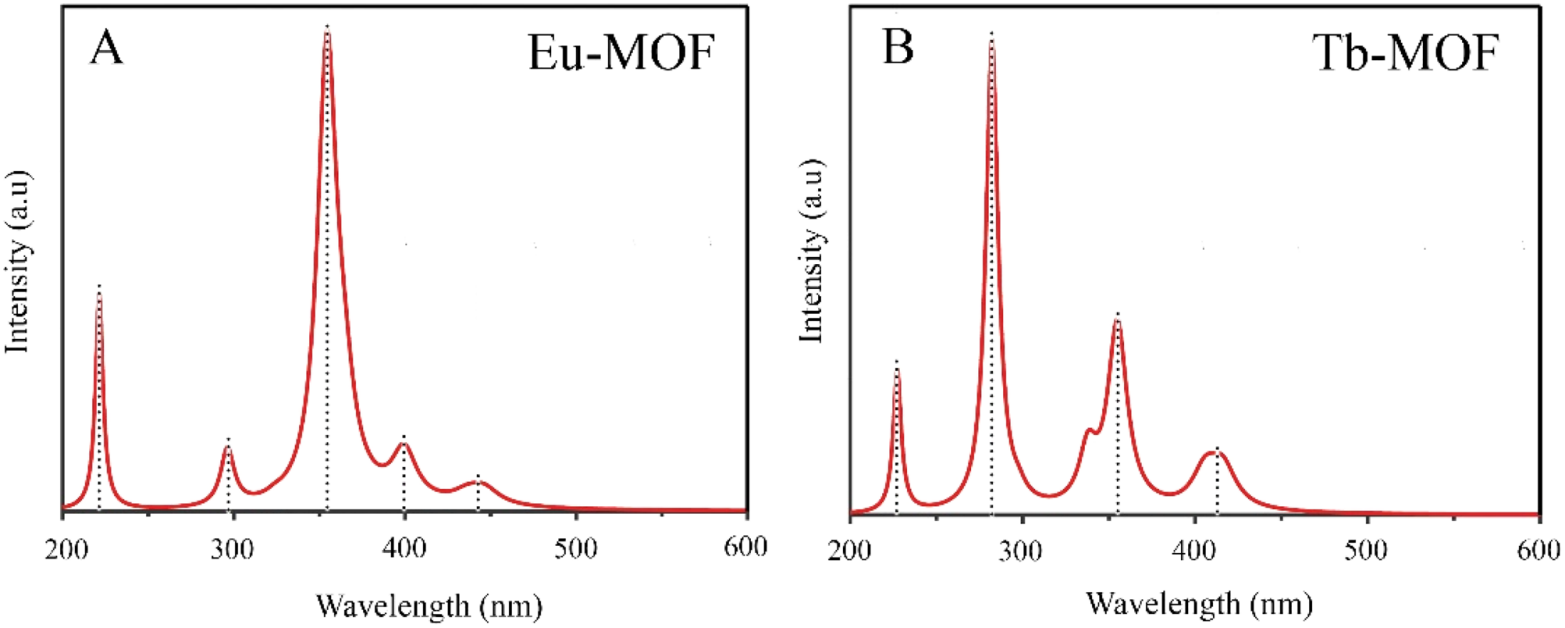
Excitation spectrum of Eu-MOF (**A**) and Tb-MOF (**B**) as a function of wavelength from DFT calculations.

The calculated lattice constants are obtained with *a* = 11.555 Å, *b* = 11.856 Å, and *c* = 11.365 Å for Eu-MOF; and *a* = 11.986 Å, *b* = 11.846 Å, and *c* = 12.979 Å for Tb-MOF. Those values are comparable to experimental results (*a* = 12.606 Å, *b* = 12.000 Å, and *c* = 12.686 Å for both Ln-MOFs). In Fig. 6, the calculated excitation spectrum of Eu-MOF and Tb-MOF are depicted as a function of wavelength. The Eu-MOF has one significant peak from absorption transitions of 355 nm, and four small peaks of 221, 295, 398, and 443 nm. Meanwhile, Tb-MOF has one significant peak at 281 nm and three small peaks at 226, 355 and 412 nm. In experiment, the excitation peaks are observed at 396 nm for Eu-MOF and 285 nm for Tb-MOF, which are comparable with theoretical results (355 nm and 281 nm for Eu-MOF and Tb-MOF, respectively).

**Figure 7:**
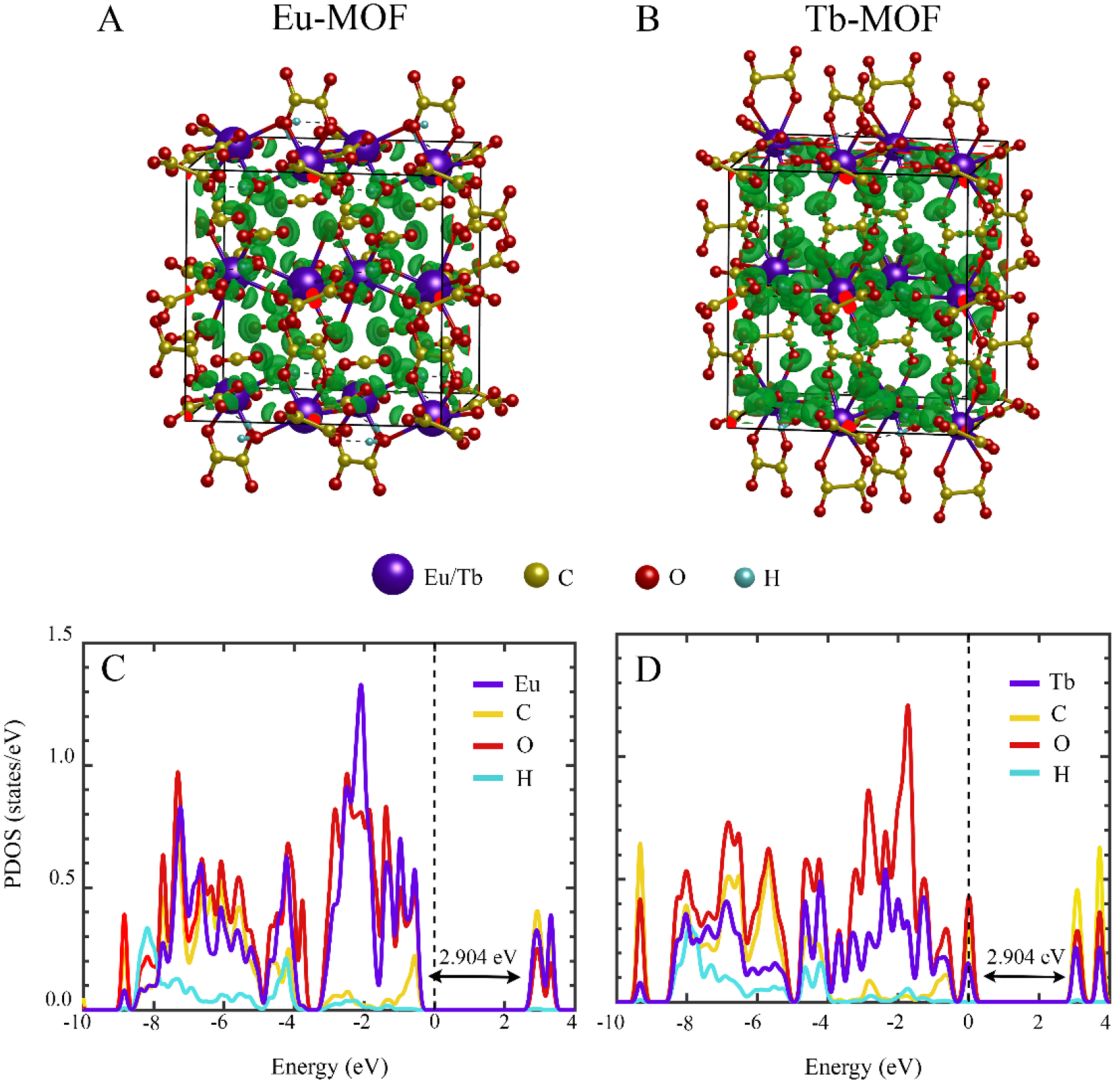
Electron localization function of Eu-MOF (**A**) and Tb-MOF (**B**). Purple sphere represents Eu or Tb; yellow, red, and cyan spheres represent C, O, and H, respectively. Partial density of states of Eu-MOF (**C**) and Tb-MOF (**D**), where the colour of lines corresponds to the colour of atoms. Fermi energy is set to zero.

In Fig. 7A,B, the electron localization function is shown to understand the bonding interactions in Eu-MOF and Tb-MOF, respectively. In these structures, the Electron Localization Function (ELF) is small between atoms indicates the ionic bonding is dominant. Overall, the maximum ELF value is observed at the O sites, while the negligible one is found at Eu/Tb, C and H sites, indicating charge-transfer interaction from Eu/Tb, C and H sites to O sites. Moreover, polarization of ELF at the O sites towards the other O sites and finite ELF between O and Eu/Tb, O and C sites indicates the hybridization interaction. Figure 7C,D show the partial density of states (PDOS) for each atom in Eu-MOF and Tb-MOF, respectively. The DOS show strong hybridization between O, Eu/Tb, and C occurs near the Fermi level (E_F_). Thus, the bonding interaction between O with Eu/Tb and C are mixed ionic-covalent character with dominant ionic bonding. It should be noted the DOS peaks of Eu-MOF is just below the E_F_ compared to one of Tb-MOF, resulting in different behaviour of electron localization function, where the ELF located around O in Eu-MOF is smaller than that in Tb-MOF. Additionally, the band gap of 2.904 and 2.597 eV are obtained for Eu-MOF and Tb-MOF, respectively.

Bader charge analysis is performed to investigate the charge-transfer interaction in Eu-MOF and Tb-MOF. Charge transfer is calculated using the following formula:

$${\text{Electron}}\;{\text{transfer}} = e_{{{\text{Neutral}}}} - e_{{{\text{Bader}}}}$$

where positive (negative) value indicates the loss (gain) electron. Table 2 shows the average charge transfer of Eu-MOF and Tb-MOF. Overall, Eu/Tb, C, and H have the positive value, while O possesses the negative value, indicating the charge transfer from Eu/Tb, C, and H sites to O sites. The results are consistent with the ELF in Fig. 7A,B, where electrons density is highly localized at O sites.

**Table 2:** Average charge transfer/atom of Eu-MOF and Tb-MOF.

Atoms	Eu-MOF	Tb-MOF
Eu/Tb	2.276	2.303
C	1.299	1.217
O	− 0.841	− 0.890
H	0.521	0.421

### Magnetic characterization of Tb-MOF

**Figure 8:**
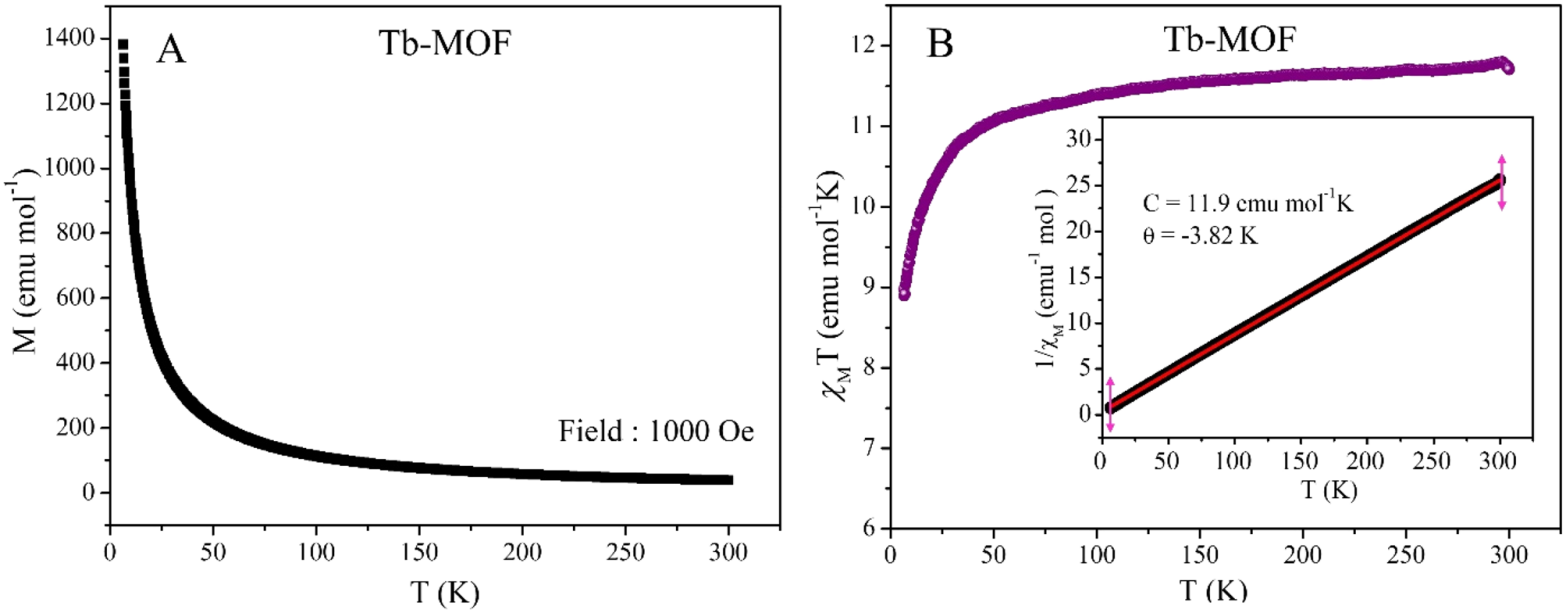
(**A**) Moment versus temperature measurement at 1000 Oe DC field. (**B**) Temperature dependence of *χ*_M_T with applied DC field of 1000 Oe from temperature range of 5–300 K. Inset shows the Curie–Weiss fitting (solid red line) of the inverse AC susceptibility.

**Figure 9:**
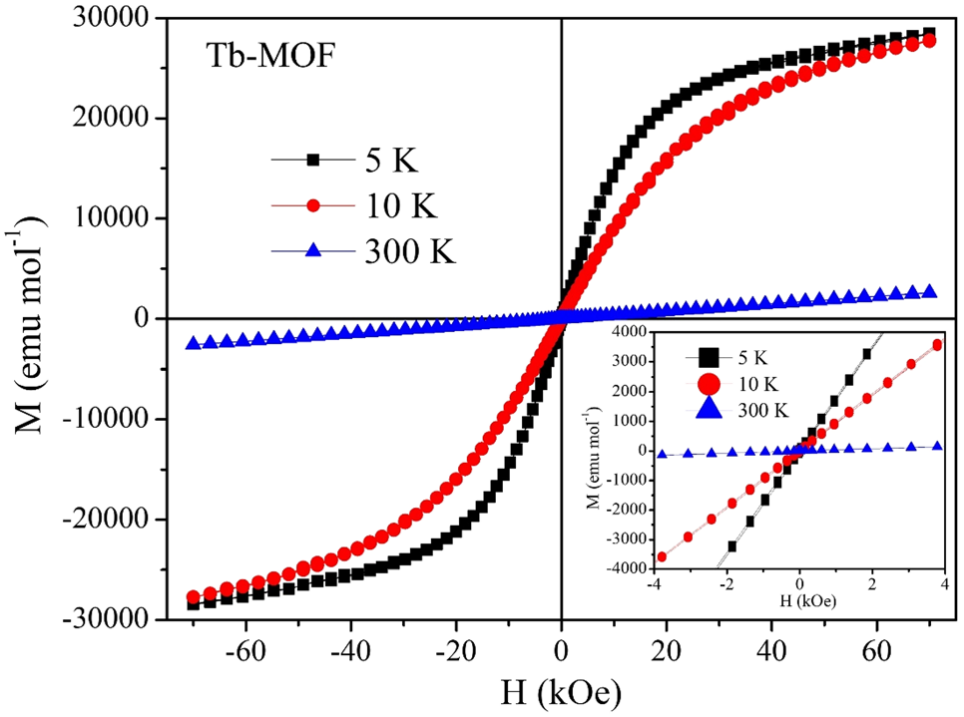
Magnetic Field dependence of Magnetization to an applied field range of − 7 T to + 7 T.

From the experimental data *χ*_M_T value at 300 K (Fig. 8) is found to be 11.70 emu mol^−1^ K. Up to 150 K the temperature dependence of *χ*_M_T is nearly constant and below 50 K it sharply decreases indicating weak antiferromagnetic interaction. From the Curie–Weiss fit the θ(K) value is found to be − 3.82 K which hint towards weak antiferromagnetism^51^. From the Curie–Weiss fit, the Curie constant C is found to be 11.90 emu mol^−1^ K and the experimentally calculated µ_eff_ is 9.75 µ_B_, which is higher than the theoretical value of 7.96 µ_B_ per Tb^3+^ ion. In Fig. 9 which shows the magnetization versus applied field measurements, there is no hysteresis loop (see inset) thus no ferromagnetic interaction is observed. The S shape MH curves with no hysteresis at 5 K and 10 K is typical for a superparamagnetic type of behavior^52,53^.

### Magnetic characterization of Eu-MOF

**Figure 10:**
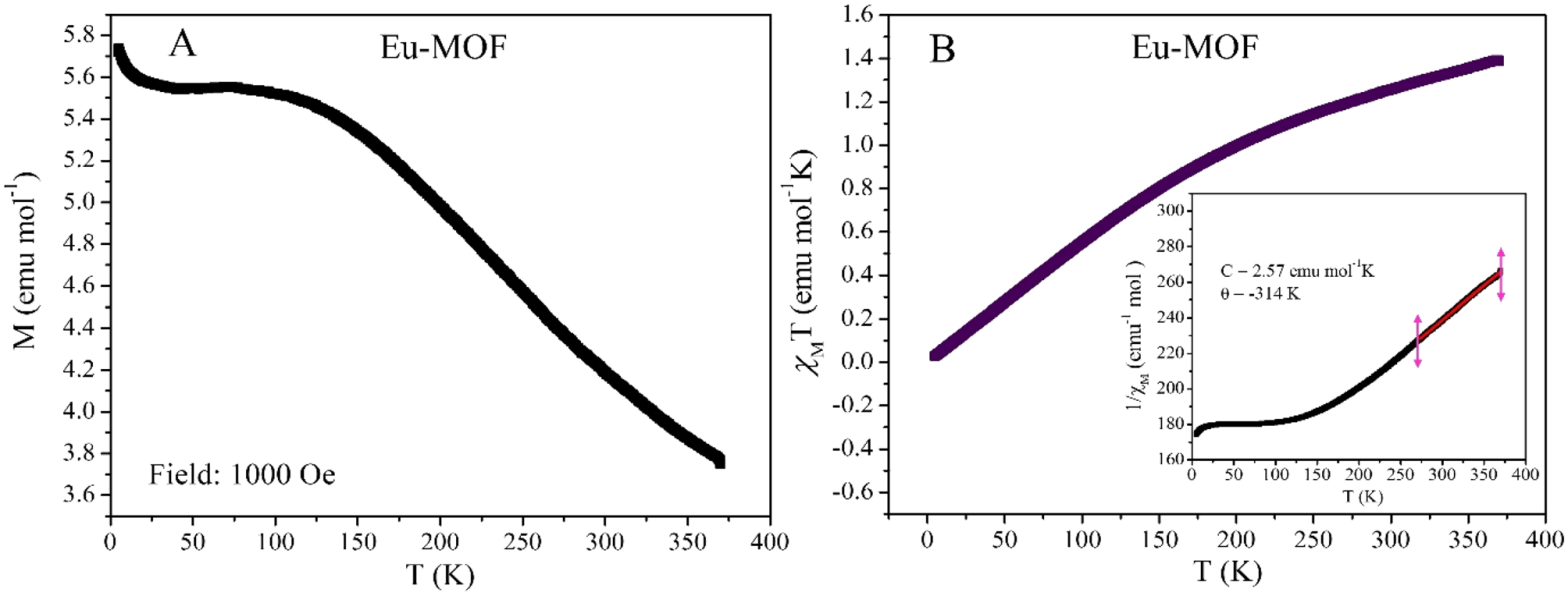
(**A**) Moment versus temperature measurement at 1000 Oe DC field. (**B**) Temperature dependence of *χ*_M_T with applied DC field of 1000 Oe from temperature range of 5–370 K. Inset shows the Curie–Weiss fitting (solid red line) at high temperature of the inverse AC susceptibility.

**Figure 11:**
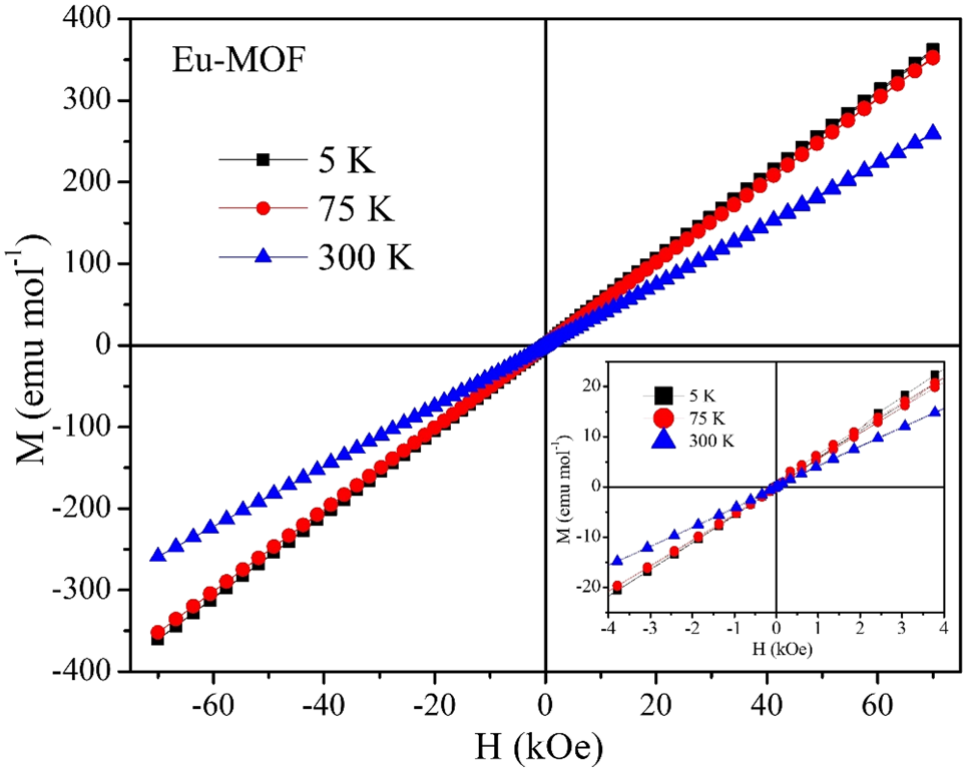
Magnetic Field dependence of Magnetization to an applied field range of − 7 T to + 7 T.

The temperature dependent DC magnetization measurements for Eu-MOF at an applied field of 1000 Oe shows dominant antiferromagnetic interaction, which is reflected in the magnetic moment versus temperature measurement as a hump near 100 K as shown in Fig. 10A. Figure 10B shows the *χ*_M_T behaviour for Eu-MOF. From the experimental data, *χ*_M_T value at 300 K is found to be 1.25 emu mol^−1^ K. The temperature dependence of *χ*_M_T is constantly decreasing as the temperature is varied from 370 down to 5 K where it reduces to 0.028 emu mol^−1^ K. The reduction in *χ*_M_T also points towards the antiferromagnetic interactions. From the Curie–Weiss fit the θ(K) value is determined to be − 308 K again signalling the antiferromagnetic interactions in the system. The Curie constant C is found to be 2.57 emu mol^−1^ K and experimentally obtained µ_eff_ is 4.50 µ_B_. From the M versus H data in Fig. 11, we see an antiferromagnetic type of behaviour at 5 K and 75 K with no hysteresis has shown in the inset.

## Conclusion

Two Lanthanide MOFs [Eu(III)- and Tb(III)-MOF] with oxalic acid linkers were hydrothermally synthesized and characterized using different analytical and spectroscopic techniques. The photoluminescent properties of these Ln-MOFs were investigated for the first time and it was shown that Eu(III) and Tb(III)-MOF are intense long-lived solid phosphorescent materials. Both metals exhibited high phosphorescence quantum yields compared to other reported luminescent MOFs. Moreover, time-resolved photoluminescent decay has indicated long-lived species of both Ln-MOFs because of the phosphorescence transition from the triplet T_1_ state to the S_0_ ground state of Ln^3+^. SQUID analysis of both Ln-MOFs has shown higher magnetic moment of Tb-MOF over Eu-MOF. Moreover, the Curie–Weiss fit of Tb-MOF shows weak antiferromagnetic interaction present at low temperatures and the magnetic field versus magnetic moment measurement reveals a superparamagnetic type of interaction at 5 K and 75 K. Eu-MOF, on the other hand, has an antiferromagnetic interaction supported by the temperature dependent magnetic moment study and the Curie- Weiss fit to the data. Similarly, analyses of magnetizations versus magnetic fields data support the argument above. This report provides full understanding of the highly photoluminescent Ln-MOFs undergoing charge transfer from the metal centre to the ligand which is studied and seen for the first time in MOF systems. These physical properties for such simply prepared robust, and inexpensive MOF materials would be of a great attraction for various fluorescence applications.

## Supplementary Information

Supplementary Information.

## Data Availability

The datasets generated during and/or analysed during the current study are included in the manuscript or uploaded as supplementary information. In addition, the datasets used and/or analyzed during the current study are available from the corresponding author on reasonable request. Crystal structures of Eu-MOF (Deposition Number: 1012765)^30^ and Tb-MOF (Deposition Number: 1913164)^27^ are available at www.ccdc.cam.ac.uk/data_request/cif.
