# Supplementary material for: A new mode of luminescence in lanthanide oxalates metal–organic frameworks
Source: Sci Rep. 2022 Nov 5;12:18812. doi: 10.1038/s41598-022-23658-z (PMC9637143; doi:10.1038/s41598-022-23658-z)
Supplement: Supplementary file 1 — Supplementary Information. [file 41598_2022_23658_MOESM1_ESM.docx]

**Supporting Information**

A New Mode of Luminescence in Lanthanide Oxalates Metal−Organic Frameworks

Reem H. Alzard,^a^ Lamia A. Siddig,^a^ Na’il Saleh,^a^ Ha L. Nguyen,^b,c^ Quynh Anh T. Nguyen,^d^ Thi H. Ho,^d^ Viet Q. Bui,^d^ K. Sethupathi,^e^ P. K. Sreejith,^e^ and Ahmed Alzamly^a,*^

^a^Department of Chemistry, UAE University, P.O. Box 15551, Al-Ain, UAE

^b^Department of Chemistry, University of California Berkeley; Kavli Energy Nanoscience Institute at UC Berkeley; and Berkeley Global Science Institute, Berkeley, California 94720, United States

^c^Joint UAEU−UC Berkeley Laboratories for Materials Innovations, United Arab Emirates University, Al-Ain 15551, United Arab Emirates

^d^Advanced Institute of Science and Technology, The University of Danang, 41 Le Duan, Danang, Vietnam

^e^Department of Physics, Low Temperature Physics Laboratory, Indian Institute of Technology Madras, Chennai 600036, India

^*^E-mail address: ahmed.alzamly@uaeu.ac.ae (A.A.)

**TABLE OF CONTENTS**

**Part 1: Characterization of Ln-MOFs……………………...…………………….…….…..…S3**

Scanning electron microscope (SEM)……...……………………………………………S3

Energy-dispersive X-ray spectroscopy (EDX)…………...……..……………………….S4

Thermogravimetric analysis of the reported Ln-MOFs….…………………………..……S6

**Part 2: Photoluminescence studies…………………..…………..……………...…….....….……….S7**

Quantum yield fitting measurements using the integrating sphere method for Ln-MOFs……………………………………………………………………...……………..S7

**Part 1: Characterization of Ln-MOFs**

Scanning electron microscope (SEM)

*
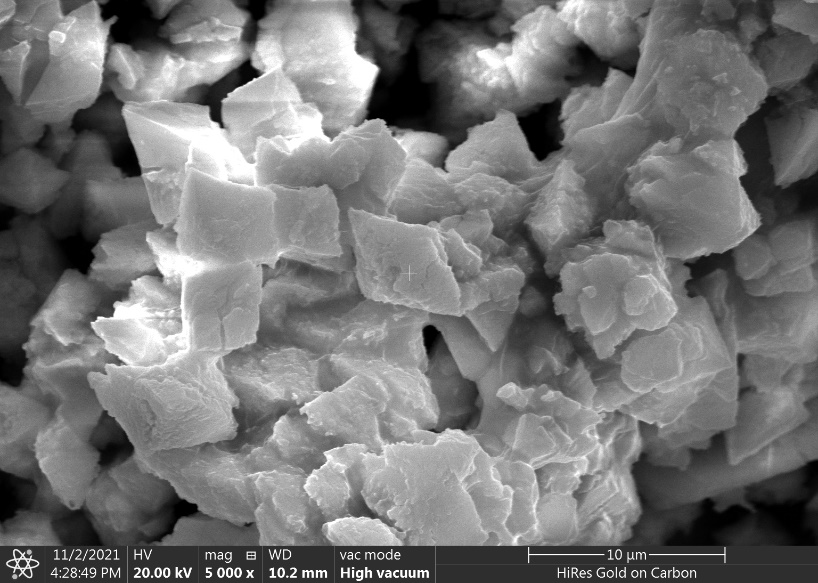
*

A

*
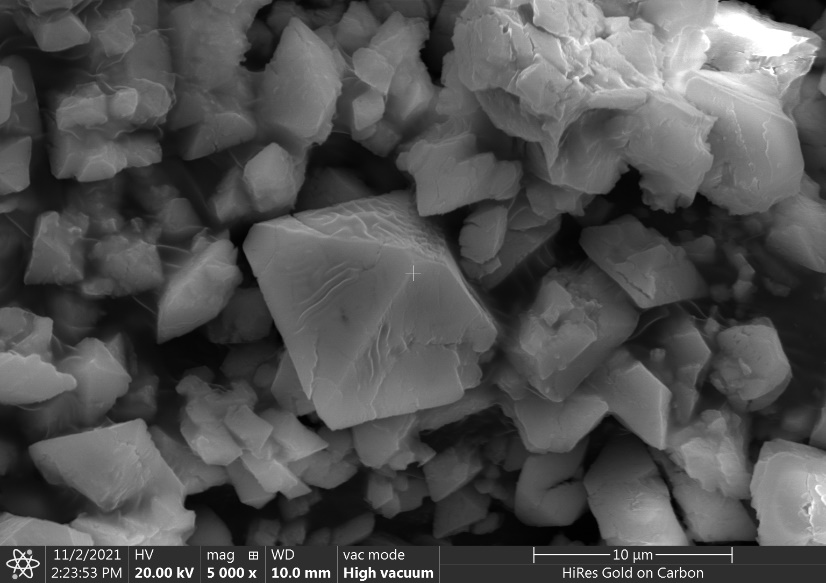
*

B

Fig. S1. SEM images of crystals of Eu-MOFs (A) and Tb-MOF (B) at 10 µm high resolution.

Energy-dispersive X-ray spectroscopy (EDX)


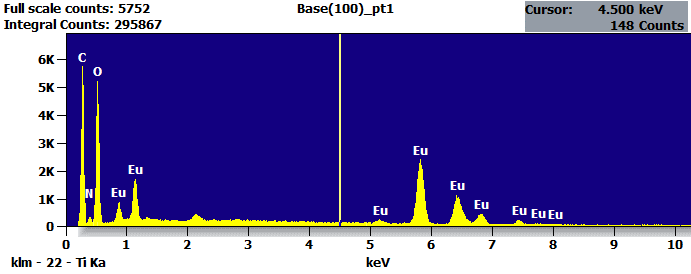


Fig. S2. EDX spectrum of Eu-MOF indicating the presence of Eu, N, O, and C atoms in the sample.

Table S1. Atomic percentages of Eu-MOF based on EDX analysis.

| Element | Weight % | Atom % |
| --- | --- | --- |
| C | 15.11 | 38.28 |
| O | 22.44 | 42.68 |
| Eu | 59.14 | 11.84 |

*
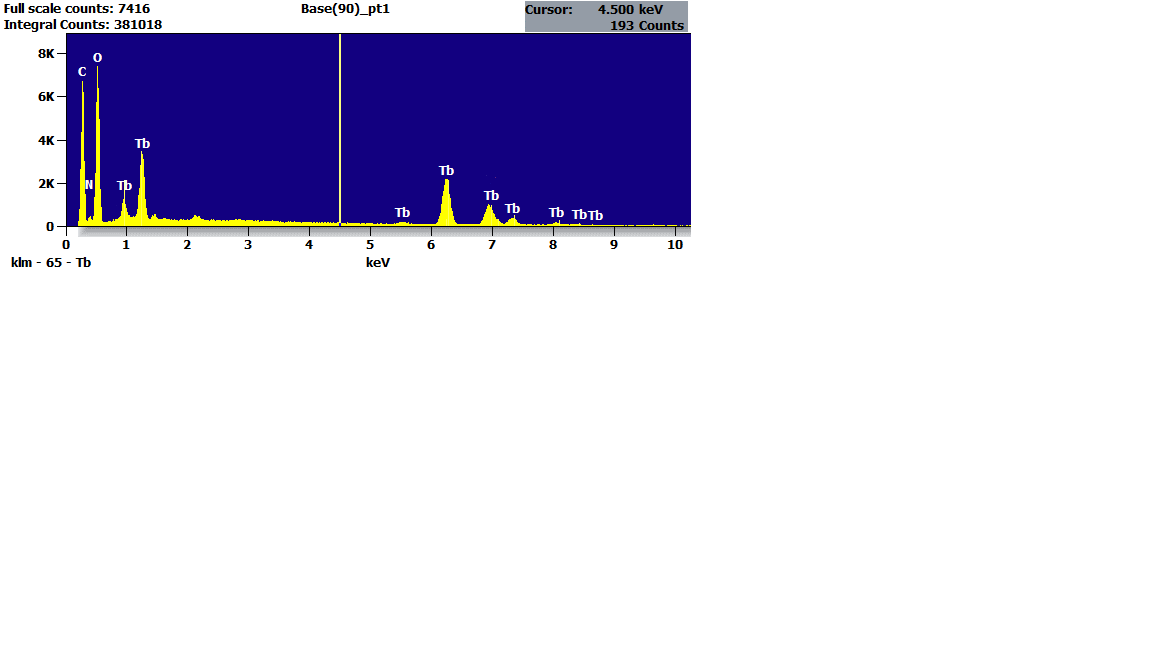
*

Fig. 3. EDX spectrum of Tb-MOF indicating the presence of Tb, N, O, and C atoms in the sample.

Table S2. Atomic percentages of Tb-MOF based on EDX analysis.

| Element | Weight % | Atom % |
| --- | --- | --- |
| C | 14.02 | 34.55 |
| O | 27.09 | 50.11 |
| Tb | 56.61 | 10.54 |

Thermogravimetric analysis of the reported Ln-MOFs


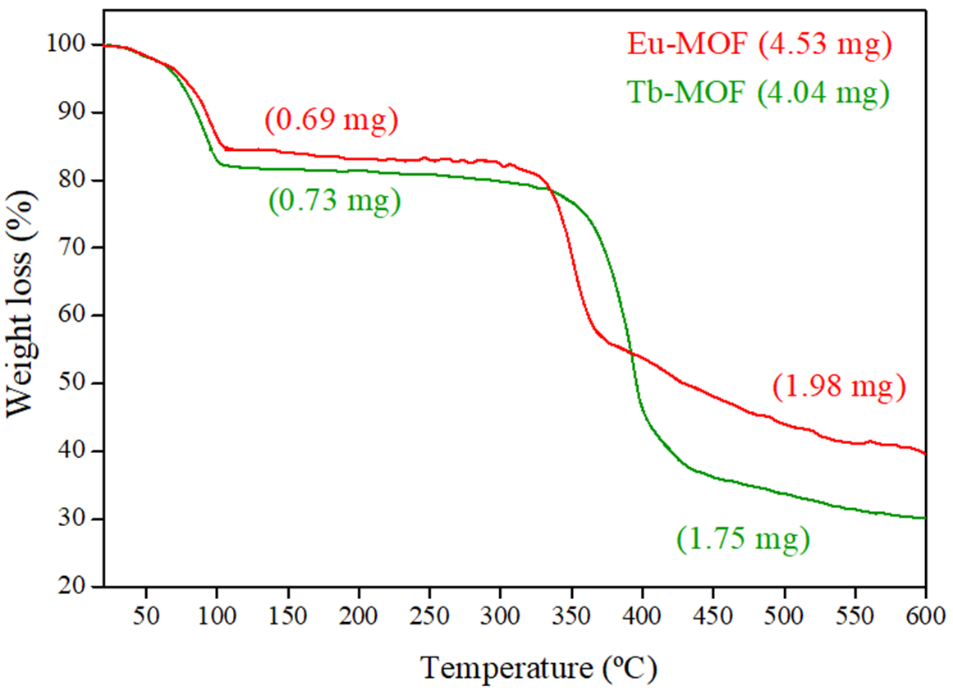


Fig. S4. Thermogravimetric analysis of Ln-MOFs with their initial masses and final masses after the thermal decomposition.

**Part 2: Photoluminescence studies**

Quantum yield fitting measurements using the integrating sphere method for Ln-MOFs


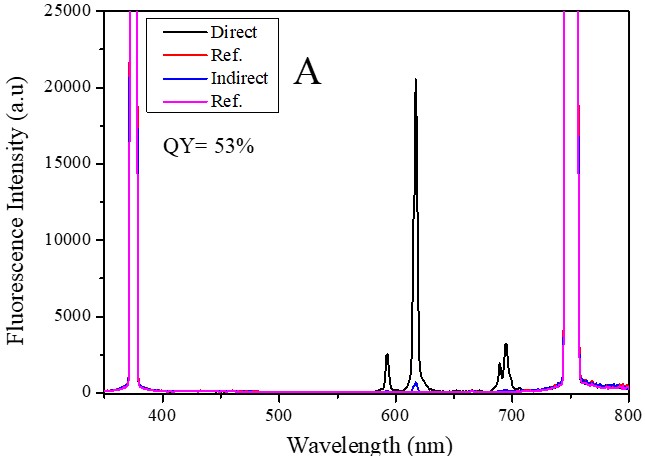


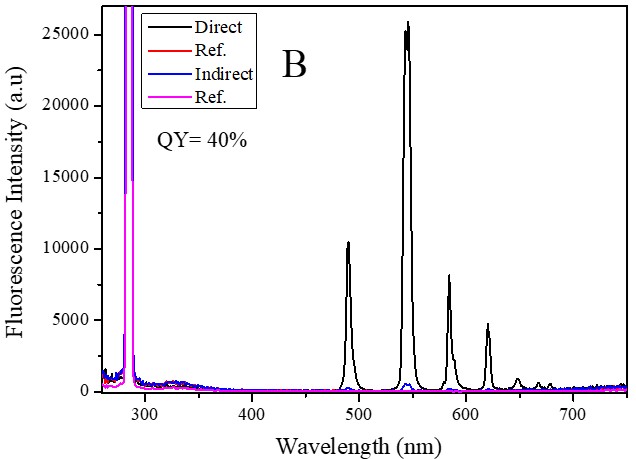


Fig. S5. Quantum yield fitting measurements using the integrating sphere method for (A) Eu-MOF and (B) Tb-MOF.
